# Supplementary material for: Local and Systemic Response to Heterogeneous Sulfate Resupply after Sulfur Deficiency in Rice
Source: Int J Mol Sci. 2022 May 31;23(11):6203. doi: 10.3390/ijms23116203 (PMC9181796; doi:10.3390/ijms23116203)
Supplement: Supplementary file 1 [file ijms-23-06203-s001.zip › Supplemental Figures.pdf]

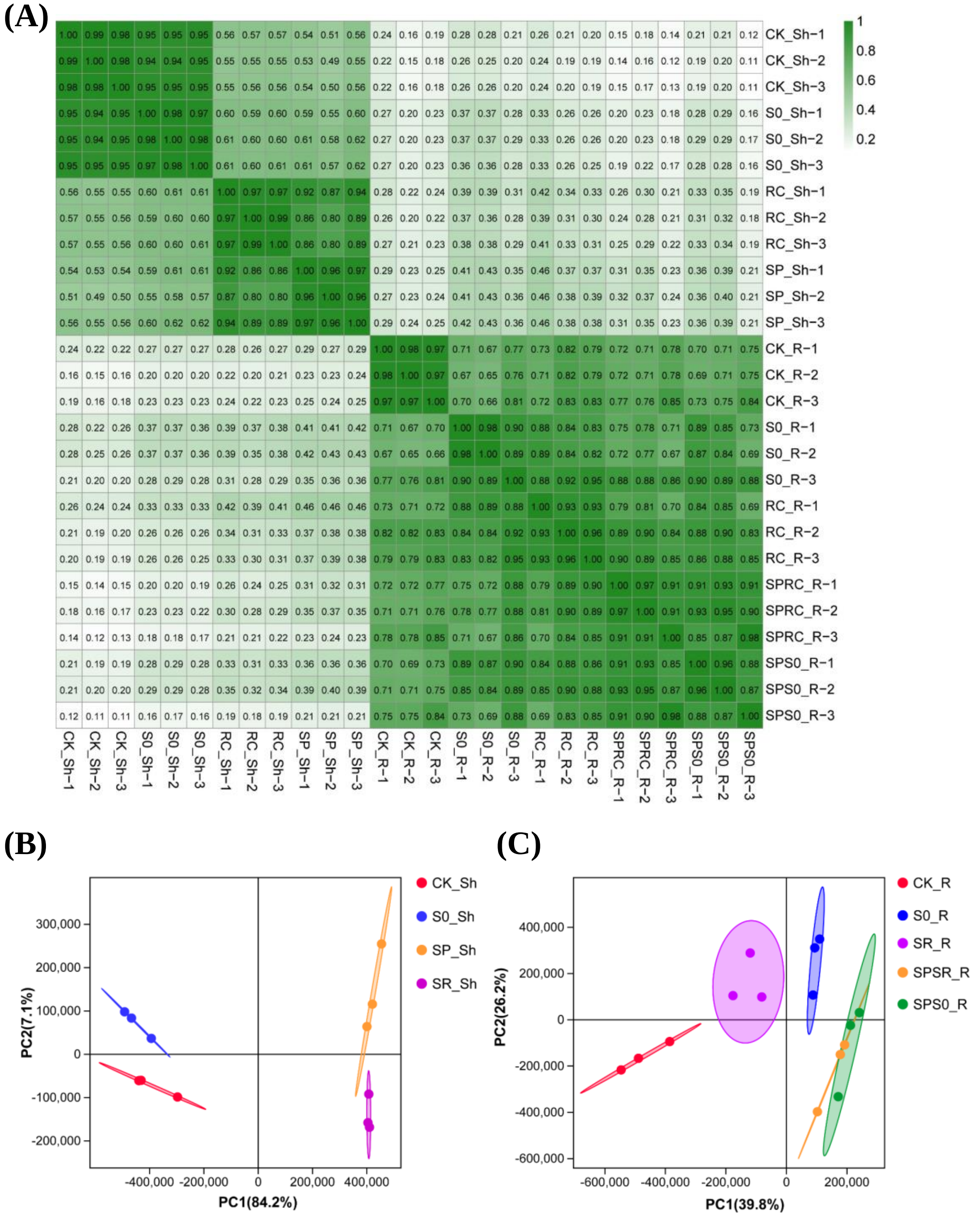

Figure S1. Pearson‘s correlation coefficient and principle component analysis (PCA) of RNA-seq samples. **(A)** Pearson‘s correlation coefficient between each samples. **(B)** PCA of shoot samples for RNA-seq. **(C)** PCA of root samples for RNA-seq.

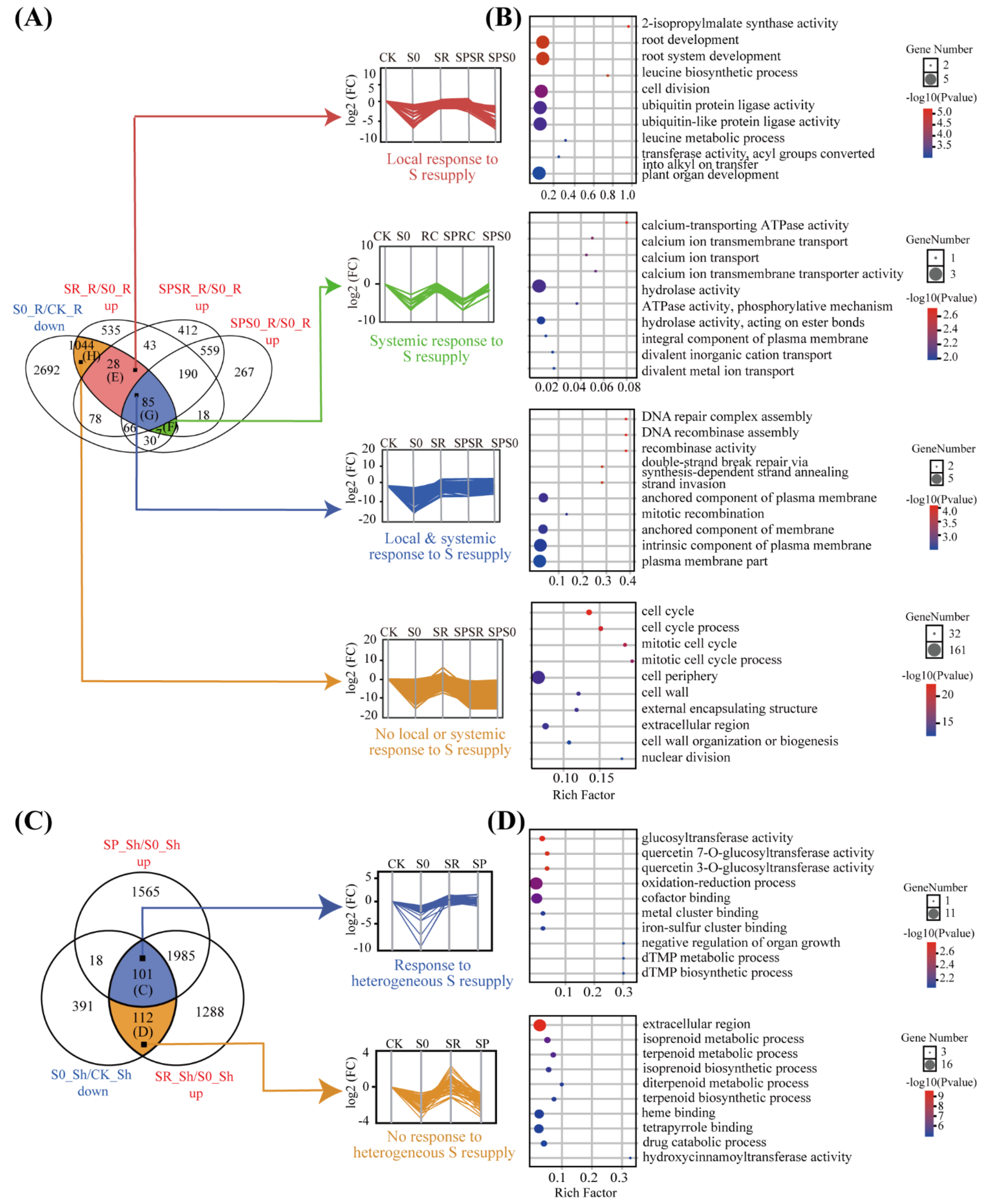

Figure S2. Root and shoot down-regulated DEGs in response to homogeneous and heterogeneous sulfate resupply. (**A**) Identification of root down-regulated DEGs with local response, systemic response, simultaneous local and systemic response and no response to sulfate resupply by gene expression trend analysis and Venn diagramming. Different types of response genes were classified into Gene set E, F, G, and H, respectively, as shown in Table S5. (**B**) GO enrichment analysis of different types of response genes in (**A**). The top 10 GO terms with lowest P-values were showed. (**C**) Identification of shoot down-regulated DEGs responding to homogeneous and heterogeneous sulfate resupply, which were classified into Gene set C and D as shown in Table S6. (**D**) GO enrichment analysis of different types of response genes in (**C**). The top 10 GO terms with lowest P-values were showed. CK, control; S0, S deficiency; SR, sulfate resupply in both sides of split-roots; SPSR, the split-root halve with sulfate resupply; SPS0, the split-root halve remained in S deficiency. Sh, shoot; R, root.
